# Supplementary material for: Microbiota in different digestive tract of paddlefish (Polyodon spathula) are related to their functions
Source: PLoS One. 2024 May 17;19(5):e0302522. doi: 10.1371/journal.pone.0302522 (PMC11101114; doi:10.1371/journal.pone.0302522)
Supplement: Attachment — Submitted filename: Comments.doc [file pone.0302522.s001.doc]

**Comments**

This study investigated the microbial diversity and community structure in intestine, stomach, and mouth of paddlefish using high-throughput sequencing, which provide a microbiological basis for the development of aquaculture. However, there are still some problems worth further discussion and revision. Details are given below.

（1）The title should be revised, since the article only conducted microbial sequencing and did not measure its function.

（2）I don't quite understand: Line 100 “...with body weight 1967.2-2471.4 g... ” , but Line 102 “...the stocking specification was 350-400 g.”, what does it refer to?

（3）The sampling time is only one, why does it take one year for breeding? Where is the meaning?

（4）There are only 5 samples, not enough.

（5）The standard deviations of the table 2 and 3 data are very large, why?

（6）Line 202: “..644 genera were detected in YC of paddlefish. ”, should be “...644 genera were detected in YZ of paddlefish.”.

（7）Line 248-249: “These research results were the best evidence for the high body fat content in the paddlefish.”, this sentence is inappropriate, so suggested to delete it.

（8）Line 255-257: “Clostridium can antagonize a variety of pathogenic fungi and also promote nitrogen accumulation (Qian et al., 2018), which will provide evidence for the water quality optimization function of paddlefish.”, this sentence is also not very accurate, it is recommended to modify it.
